# SEVIRI Hyper-Fast Forward Model with Application to Emissivity Retrieval

**DOI:** 10.3390/s19071532

**Published:** 2019-03-29

**Authors:** Guido Masiello, Carmine Serio, Sara Venafra, Laurent Poutier, Frank-M. Göttsche

**Affiliations:** 1 School of Engineering, University of Basilicata, 85100 Potenza, Italy; carmine.serio@unibas.it (C.S.); sara.venafra@unibas.it (S.V.); 2 ONERA, The French Aerospace Lab, 2 avenue Edouard Belin, 31055 Toulouse Cedex, France; laurent.poutier@onera.fr; 3 Karlsruhe Institute of Technology, IMK-ASF, Hermann-von-Helmholtz-Platz 1, 76344 Eggenstein-Leopoldshafen, Germany; frank.goettsche@kit.edu

**Keywords:** fast forward model, infrared, emissivity spectrum, satellite, validation

## Abstract

Timely processing of observations from multi-spectral imagers, such as SEVIRI (Spinning Enhanced Visible and Infrared Imager), largely depends on fast radiative transfer calculations. This paper mostly concerns the development and implementation of a new forward model for SEVIRI to be applied to real time processing of infrared radiances. The new radiative transfer model improves computational time by a factor of ≈7 compared to the previous versions and makes it possible to process SEVIRI data at nearly real time. The new forward model has been applied for the retrieval of surface parameters. Although the scheme can be applied for the simultaneous retrieval of temperature and emissivity, the paper mostly focuses on emissivity. The inverse scheme relies on a Kalman filter approach, which allows us to exploit a sequential processing of SEVIRI observations. Based on the new forward model, the paper also presents a validation retrieval performed with in situ observations acquired during a field experiment carried out in 2017 at Gobabeb (Namib desert) validation station. Furthermore, a comparison with IASI (Infrared Atmospheric Sounder Interferometer) emissivity retrievals has been performed as well. It has been found that the retrieved emissivities are in good agreement with each other and with in situ observations, i.e., average differences are generally well below 0.01.

## 1. Introduction

SEVIRI (Spinning Enhanced Visible and Infrared Imager) is the moderate resolution imager on board the Meteosat Second Generation (MSG) operated by EUMETSAT (European Centre for the Exploitation of Meteorological Satellites), which provides image data in four visible and near infrared channels and eight thermal infrared channels. These twelve different spectral channels provide the capability of cloud imaging and tracking, fog detection, measuring o Earth surface and cloud top temperatures, tracking ozone patterns, as well as many other features. A new image of Earth disk is provided every 15 min. The sampling distance is about 3 km for the infrared and three visible channels, and 1 km for the high-resolution visible channel.

SEVIRI is onboard a geostationary platform and as such its observations can resolve the diurnal cycle with high temporal resolution. There is evidence that time–space constraints can significantly enhance our ability to extract information from geostationary data in comparison to ‘single-pixel’ algorithms which only use the spectral information [1,2,3]. Hence there is a need to explore SEVIRI’s full observational space to improve the quality of operationally derived products.

In a series of recent papers [1,2,3], the authors have described and presented a general Kalman Filter methodology for the simultaneous retrieval of surface emissivity (ε) and temperature ($T_{s}$) from SEVIRI infrared radiances. The KF or Kalman filter (e.g., see [4,5]) provides a general framework to develop physically based retrieval algorithms, which can exploit the temporal continuity expected from geostationary instruments such as SEVIRI. The KF approach was applied for the first time to the combined retrieval of ε, $T_{s}$ by [1] and further improved and validated by [2,3]. Physically based schemes for the same problem and instrument have been also considered and developed by [6,7], although in this case the retrieval is based on a static Optimal Estimation approach.

The retrieval of surface emissivity and temperature from space has been predominantly focus on statistical approaches and we refer the interested reader to [8,9] for reviews. Apart from SEVIRI, physically based ε, $T_{s}$ retrieval algorithms have been considered by various authors for applications to imagers and hyper-spectral infrared sounders (among many other we quote [10,11,12,13,14,15,16]).

One of the most important aspects when dealing with a physically based retrieval algorithms is the use of an effective and, possibly, fast forward model. It has to be stressed that with SEVIRI the number of observations is of order $10^{8}$ per 15 min and will increase of a factor 100 or more with Meteosat Third Generation (see, e.g., [17]).

For the class of instruments, such as SEVIRI, which have channels with a relatively large bandwidth, the forward model can be appropriately simplified in order to gain computational efficiency. The usual approach is to rely on generic band models, such as, e.g., MODTRAN (MODerate resolution atmospheric TRANsmission) [18]. Conversely, our approach is to build a customized forward model for SEVIRI and develop it considering that the model has to be used in the inverse methodology. In this case, the forward model is specifically optimized for SEVIRI and emissivity retrieval, although the methodology could be used for other multi and hyperspectral imaging sensors.

The main objective of this work is to present and discuss a new approach to develop a forward model for SEVIRI, which is intended for the retrieval of emissivities at three infrared channels: 8.7, 10.8 and 12.0 μm, along with surface temperature. The new approach is based on the basic methodology already developed in [19,20] and considers two additional steps, which make use of a suitable selection of radiance predictors along with the well known Principal Component Analysis (PCA) transform to further reduce the dimensionality of the spectral radiance data space. The new forward model has been embedded within our KF scheme [1,2], and a retrieval exercise has been performed to show its effectiveness in retrieving emissivities. The retrieval exercise presented here has been performed over highly homogeneous and flat gravel plains around the permanent land surface temperature (LST) validation station *Gobabeb* in the Namib desert [16,21,22] operated by Karlsruhe Institute of Technology (KIT) within the framework of EUMETSAT’s Satellite Application Facility on Land Surface Analysis (LSA SAF).

Although the paper can simultaneous retrieve surface temperature and emissivity, the application shown in this paper is focused on emissivity. In effect, a comprehensive retrieval exercise for the validation of surface temperature has been already performed using in situ surface temperature observations [2]. For the Gobabeb validation station, based on one year long dataset of hourly surface temperature data, we estimated a root mean square error of ≈1.26 K [2].

The paper is organized as follow. Section 2 is devoted to the description of the new forward model, Section 3 will describe data used for the validation exercise, Section 4 will show the results of the retrieval/validation exercise, while conclusions will be drawn in Section 5.

## 2. Principal Component Analysis Approach to SEVIRI Radiative Transfer Modelling

This section describes the methodology developed to compute radiance and its derivatives with respect to surface parameters at SEVIRI instrument spectral resolution.

SEVIRI’s eigth infrared channels are listed in Table 1. The methodology has been specifically developed, optimized and tested for the three atmospheric window infrared channels: 8.7, 10.8, 12 μm, which are the channels for which we can retrieve emissivity. Channel 4 is contaminated by solar radiation during the day and, therefore, not considered for retrieval. Channels 5 and 6 are strongly affected by water vapour absorption and cannot see the surface. Channel 8 is contaminated by ozone, whereas channel 11 lies in the CO${}_{2}$ longwave absorption band and is also contaminated by ice causing corresponding observation be biased.

The methodology we have developed needs a monochromatic forward model for the *training* phase. To this end we use the monochromatic forward model that we call σ-IASI [23,24,25,26]. The forward model optimized for SEVIRI is based on a previous version [19,20], specifically developed for surface emissivity and temperature. In the rest of this paper, the old version will be referred to as σ-SEVIRI, whereas the new one as $\sigma_{pc}$-SEVIRI.

### 2.1. Radiative Transfer Modelling

At SEVIRI spectral resolution, the top of the atmosphere (TOA) radiance in a given channel receives contributions from thousands of gas absorption lines. In fact, depending on air pressure, the line spacing can be as fine as $10^{-4}$ cm^−1^. However, because of the high spectral redundancy, it is expected that the sampling can be optimized in order to reduce the monochromatic radiance data space. This approach was taken to develop the previous version of the forward module or σ-SEVIRI. A look-up table of monochromatic optical depth was developed, with a sampling of $10^{-2}$ cm^−1^, which formed the basis of fast SEVIRI radiance computation [19,20]. The use of a monochromatic look-up table gives efficiency to the fast forward modelling, especially when considering the generation of radiances at off-nadir viewing angles. Furthermore, the model can be applied to other instruments just by changing the appropriate Instrument Spectral Response Function or ISRF.

The new $\sigma_{pc}$-SEVIRI retains the formalism of the monochromatic look-up table, but it attempts to further reduce the dimensionality of the data space based on the assumption that any given SEVIRI TOA radiances can be represented as a function of only few monochromatic quantities or predictors. In this way, we can save storage and computational time compared to the previous version, as we will show in the rest of this section.

To begin with, let us consider the radiative transfer equation in a form which is suitable for modelling the atmospheric window SEVIRI channels in infrared. The monochromatic spectral resolution TOA radiance, R(σ) at wave number σ can be written according to

(1) $$R\left(\sigma \right)=\varepsilon \left(\sigma \right)\tau_{0}\left(\sigma \right)B\left(T_{s};\sigma \right)+A\left(\sigma \right)+\left(1-\varepsilon \right)\tau_{0}F\left(\sigma \right)$$

where, to shorten notation, we have considered implicitly the dependence of the various terms on the viewing angle or satellite Field of View (FOV). In Equation (1),

- ε(σ) is the surface emissivity
- $\tau_{0}\left(\sigma \right)$ is the total atmospheric transmittance
- $B(T_{s};\sigma )$ is the Planck function computed at the surface temperature, $T_{s}$
- A(σ) is the atmospheric emission term
- F(σ) is the down-welling thermal radiation reflected at the surface within the satellite viewing angle

Unless needed, in the rest of this section, we will omit the dependence of the various terms in Equation (1) on the wave number, σ.

The up-welling atmospheric emission term can further decomposed in

(2) $$A=\int_{0}^{\infty }B\left(T\left(z\right)\right)\frac{\partial \tau (z)}{\partial z}dz$$

with τ(z) the atmospheric transmittance from level *z* to *∞*. Furthermore, down-welling radiance term can be written as

(3) $$F=\int_{\infty }^{0}B\left(T\left(z\right)\right)\frac{\partial \tau^{*}\left(z\right)}{\partial z}dz;$$

with (see, e.g., [13])

(4) $$\tau^{*}=\left\{\begin{array}{cc} \mathrm{the}\;\mathrm{transmittance}\;\mathrm{from}\;\infty \;\mathrm{to}\;\mathrm{level}z; & \mathrm{For}\;a\;\mathrm{specular}\;\mathrm{surface}\\ \mathrm{the}\;\mathrm{slab}\;\mathrm{or}\;\mathrm{diffuse}\;\mathrm{trasnmittance}; & \mathrm{For}\;a\;\mathrm{Lambertian}\;\mathrm{surface} \end{array}\right.$$

Equation (1) can be re-arranged as

(5) $$R=\tau_{0}B\left(T_{s}\right)+A+\left(1-\varepsilon \right)\tau_{0}\left(F-B\left(T_{s}\right)\right)$$

with the substitutions

(6) $$I_{0}=\tau_{0}B\left(T_{s}\right)+A$$

and

(7) $$D=\tau_{0}\left(F-B\left(T_{s}\right)\right)$$

Equation (5) becomes

(8) $$R=I_{0}+\left(1-\varepsilon \right)D$$

Based on Equations (5) and (8), the derivative of *R* with respect to surface emissivity and temperature can be easily obtained,

(9) $$\frac{\partial R}{\partial \varepsilon }=-D$$

(10) $$\frac{\partial R}{\partial T_{s}}=\varepsilon \tau_{0}\frac{\partial B(T_{s})}{\partial T_{s}}$$

Let Q(σ) be a generic function of wave number σ. If we want to compute Q(σ) at SEVIRI spectral resolution, we have to convolve it with the appropriate Instrumental Spectral Response Function (ISRF). For a given channel with center $\sigma_{0}$, we have

(11) $$\langle Q\left(\sigma_{0}\right)\rangle =\int_{0}^{+\infty }\mathrm{ISRF}\left(\sigma -\sigma_{0}\right)Q\left(\sigma \right)d\sigma$$

where the angular brackets denote “computed at SEVIRI spectral resolution”. With this in mind, we have for the radiance and its derivatives

(12) $$\langle R\rangle =\langle I_{0}\rangle +\left(1-\bar{\varepsilon }\right)\langle D\rangle$$

(13) $$\frac{\partial \langle R\rangle }{\partial \bar{\varepsilon }}=-\langle D\rangle$$

(14) $$\frac{\partial \langle R\rangle }{\partial T_{s}}=\bar{\varepsilon }\langle \tau_{0}\frac{\partial B(T_{s})}{\partial T_{s}}\rangle \approx \bar{\varepsilon }\langle \tau_{0}\rangle \left(b_{1}{\left.\frac{\partial B(T_{s})}{\partial T_{s}}\right|}_{\sigma_{0}}+b_{0}\right)$$

where $\bar{\varepsilon }$ is the *effective channel* emissivity, which, based on the mean theorem, is given by

(15) $$\bar{\varepsilon }=\frac{\int_{0}^{+\infty }\varepsilon D\left(\sigma \right)\mathrm{ISRF}\left(\sigma -\sigma_{0}\right)d\sigma }{\int_{0}^{+\infty }D\left(\sigma \right)\mathrm{ISRF}\left(\sigma -\sigma_{0}\right)d\sigma }$$

Furthermore, in Equation (14) the two coefficients $b_{0},b_{1}$ are determined so that for each channel, $\sigma_{0}$ they minimize the following relation

(16) $$\left|\langle \tau_{0}\frac{\partial B(T_{s})}{\partial T_{s}}\rangle -\langle \tau_{0}\rangle \left(b_{1}{\left.\frac{\partial B(T_{s})}{\partial T_{s}}\right|}_{\sigma_{0}}+b_{0}\right)\right|$$

From Equations (12)–(14), we see that the radiance and its derivative with respect to the surface parameters at instrument resolution can be evaluated as a function of the three convoluted terms: $\langle I_{0}\rangle$, 〈D〉, and $\langle \tau_{0}\rangle$. According to Equation (11), the exact calculations of these three terms relies on a line-by-line or monochromatic radiative transfer model for the computation of the given function at infinite spectral resolution. However, this is expensive and time consuming. Therefore, we try to fit the convoluted generic quantities, $\langle Q\left(\sigma_{0}\right)\rangle$ using a finite set of monochromatic values $Q\left(\sigma_{i}\right),i=1,\ldots ,n_{pr}$, with $\sigma_{i}$ ranging within the bandwidth of the given channel centered at $\sigma_{0}$, that is

(17) $$\langle Q\rangle \approx \hat{\langle Q\rangle }=\sum_{i=1}^{n_{pr}}a_{i}Q_{i}$$

with $a_{i}$ suitable regression coefficients to be determined and where we have written $Q_{i}$ for $Q(\sigma_{i})$ and 〈Q〉 for $\langle Q\left(\sigma_{0}\right)\rangle$. In Equation (17), $\hat{\langle Q\rangle }$ is the estimate of 〈Q〉 based on the linear regression fit.

Of course, the linear representation has to be developed for $\langle I_{0}\rangle$, 〈D〉, and $\langle \tau_{0}\rangle$. How we choose predictors for each channel and $\langle I_{0}\rangle$, 〈D〉, $\langle \tau_{0}\rangle$, respectively, will be discussed in the next subsection.

Before closing this section, we want to stress that the concept of channel emissivity we have introduced in our formalism (see Equation (15)), refers to the spectral variability of emissivity. In effect, our formulation applies to pure scene types with a single uniform temperature, therefore we expect that application to non-uniform scenes could contain biases as a function of solar zenith angle. However, it should be also stressed that for a geostationary instrument, such as SEVIRI, a given pixel is imaged any time at the same viewing angle, so that the scene geometry remains stationary. For non uniform scenes, our methodology could be extended to consider the use of a radiative transfer approach that includes models of surface variability as, e.g., discussed in [27,28,29].

### 2.2. Linear Regression and PCA Decomposition

The predictors $Q_{i},i=1,\ldots ,n_{pr}$ can be arranged in a vector, Q,

(18) $$Q={\left\{Q_{1},\ldots ,Q_{n_{pr}}\right\}}^{t}$$

where the superscript *t* indicates the transpose operation. In order to compute the regression coefficients, $a_{i}$, we need a suitable ensemble of these vectors, ${\left\{Q_{j}\right\}}_{j=1,\ldots ,m}$ or equivalently a suitable set of atmospheric state vectors, which we can input to our monochromatic radiative transfer model σ-IASI for the calculation of the monochromatic function, Q(σ). The state vector consists of surface temperature ($T_{s}$), temperature profile (*T*), H${}_{2}$O mixing ratio profile (*q*), and ozone mixing ratio profile (*o*). These are the atmospheric parameters which mostly govern the TOA SEVIRI radiance within the atmopsheric window channels. The state vectors are derived from the well known Chevalier database [30]. We derived 100 clear sky state vectors which are uniformly distributed across the SEVIRI full disk, but at viewing angles below 70${}^{\circ }$. Another set of 100 state vectors was selected for validation. It should be stressed that we do not need to consider the natural variability of surface emissivity when building the training and validation data sets, because $\langle I_{0}\rangle$, 〈D〉, and $\langle \tau_{0}\rangle$ are independent of ε. The surface emissivity is an user defined parameter. This is a big advantage of our forward modelling approach, which would have been lost if we had parameterized the calculation directly in terms of SEVIRI radiances.

**Figure 1:**
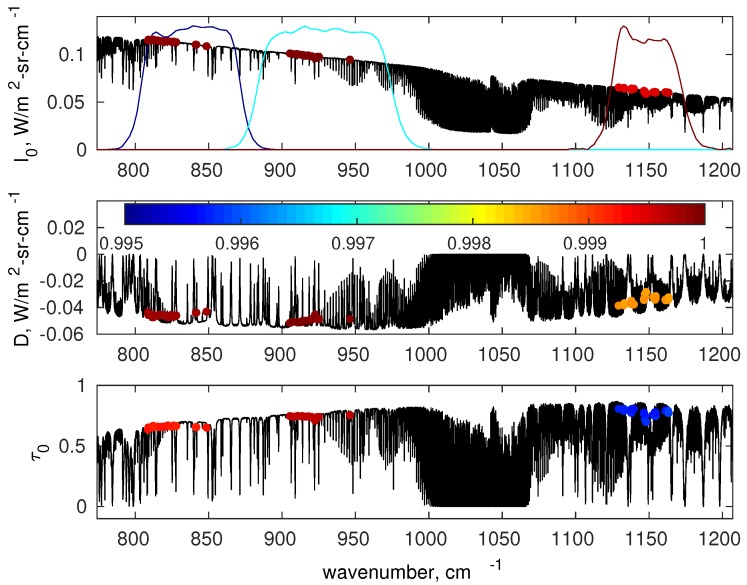
Example for the selection of monochromatic predictors $I_{0}$ (**top** panel), *D* (**middle** panel) and $\tau_{0}$ (**bottom** panel). The shown monochromatic predictors exemplify a tropical sate vectors over the spectral range 750 to 1200 cm${}^{-1}$. The position of the $n_{pr}$ predictors are indicated with coloured circles; the colour scale shows the linear correlation coefficient between SEVIRI channel quantities ($\langle I_{0}\rangle$, 〈D〉 and $\langle \tau_{0}\rangle$) and the single monochromatic predictor. In the top panel coloured lines indicate to the ISRF of the three SEVIRI channels used in this work.

The criterion to select the predictor channels relies on the maximization of the linear correlation coefficients between the single channel and the correct value of 〈Q〉. The number of predictors for each SEVIRI channel is summarized in Table 2, whereas their spectral position is shown in Figure 1. We stress that the predictors position is the same for $\langle I_{0}\rangle$, 〈D〉, and $\langle \tau_{0}\rangle$. For each given monochromatic wave number, we have three correlation coefficients corresponding to $\langle I_{0}\rangle$, 〈D〉, and $\langle \tau_{0}\rangle$, respectively. We chose the minimum of these correlation coefficients; then, for each SEVIRI channel, we chose the wave number positions with the larger correlation coefficients. We have two other conditions to choose the final set of predictor positions. The first one is still local: the corresponding correlation coefficient has to be greater than 0.995; the second one is global: the root mean square error of the difference ${\langle R\rangle }_{rc}-\langle R\rangle$ has to be lower than the corresponding radiometric noise (see Table 1), where 〈R〉 and ${\langle R\rangle }_{rc}$ are the channel radiances computed according to the exact line-by-line calculation (e.g., see Equation (12)) and the reconstructed radiances according to the linear regression approach.

The regression approach can be further improved in order to reduce the dimensionality of the predictors space. To achieve this objective we resort to Principal Component Analysis.

Let X be the matrix whose columns are the predictor vectors computed on the basis of the training data set. This matrix has dimension $n_{pr}\times m$, where for each SEVIRI channel $n_{pr}$ is given in Table 2 and m=100, that is the number of state vectors in the training data set,

(19) $$X=\left(\begin{array}{ccc} Q_{1,1} & \cdots & Q_{1,m}\\ \cdots & \cdots & \cdots \\ Q_{n_{pr},1} & \cdots & Q_{n_{pr},m} \end{array}\right)$$

Based on matrix X, we compute the corresponding covariance matrix, C=cov(X), which has size $n_{pr}\times n_{pr}$. Matrix C is then decomposed through Singular Value Decomposition,

(20) $$C=USV^{t}$$

Once we have the unitary basis U, each predictor vector, Q, can be projected onto this basis,

(21) $$Q=Uc;\;\mathrm{with}\;c=U^{t}Q$$

Next we consider a truncated expansion of Q based on the first $r<n_{pr}$ principal components,

(22) $$c_{r}=U_{r}^{t}Q$$

where the matrix $U_{r}$ is made of the first *r* columns of U. In other words, the matrix $U_{r}$ has size $n_{pr}\times \tau$. Finally, the linear regression is built up in terms of the truncated PC scores vector, $c_{r}$,

(23) $$\langle Q\rangle \approx \hat{\langle Q\rangle }=\sum_{i=1}^{r}w_{i}\cdot c_{i}$$

where $w_{i}$ are the regression coefficients in PC space.

The number, *r* of PC scores retained for each SEVIRI channel is shown in Table 2.

At this point, it should be stressed that the predictor vectors, Q depend on viewing angle, θ. More precisely, the dependence is on the inverse of cos(θ) or μ=sec(θ). Since we analyse angles below 70${}^{\circ }$, μ lies in the interval [1-2.92]. The interval $\left[0^{\circ }-70^{\circ }\right]$ is divided into 14 bins of width $5^{\circ }$, $5^{\circ }\cdot \left(j-1\right)\leq \theta <5^{\circ }\cdot j,\;j=1,\ldots ,14$. To take into account the dependence of the couple ($U_{r},w$) on viewing angle, ($U_{r},w$) is computed for each θ centered on these 14 bins.

Finally, we stress that the selection of *r* for each channel is again based on the global criterion that root mean square difference ${\langle R\rangle }_{rc}-\langle R\rangle$ has to be less than the corresponding radiometric noise.

**Figure 2:**
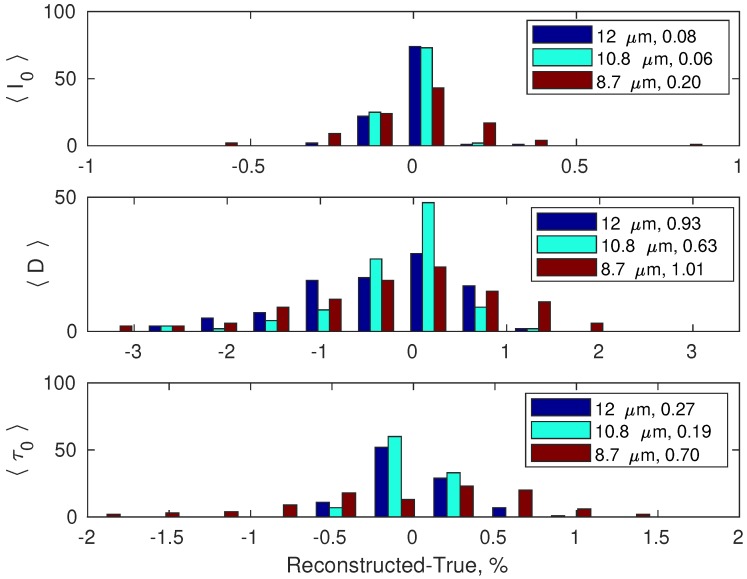
Residuals distribution (reconstructed-true) in percentage units for the three quantities $\langle I_{0}\rangle$ (upper panel), 〈D〉 (middle panel) and $\langle \tau_{0}\rangle$ (lower panel), for the three SEVIRI channels. The numbers in the legends show percentage root mean squared differences.

Figure 2 summarizes the performance of the PCA-based forward model as far as the estimation of the single channel parameters $\langle I_{0}\rangle$, 〈D〉 and $\langle \tau_{0}\rangle$ is concerned. The results are based on the validation datasetand correspond to the viewing angle first bin ($0^{\circ }\leq \theta <5^{\circ }$).

**Figure 3:**
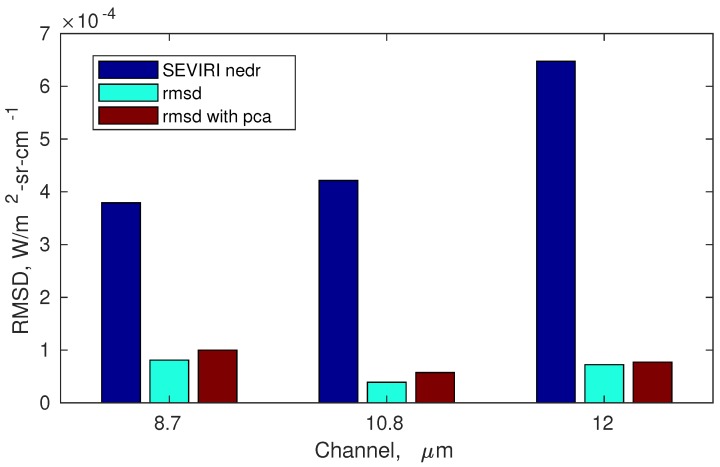
Radiance root means square difference or rmsd (reconstructed-true) for the three SEVIRI channels and comparison with the corresponding radiometric noise. The rmsd is shown for the old (σ-SEVIRI) and new version ($\sigma_{pc}$-SEVIRI) of the forward model described in this paper.

Finally, Figure 3 shows the performance of the new forward model, $\sigma_{pc}$-SEVIRI in terms of radiance root means square difference (reconstructed-true) for each SEVIRI channel. The root mean square difference has been calculated on the basis of the validation data set. Its comparison with the SEVIRI radiometric noise shows that the difference with a more accurate line-by-line calculation is well below the noise standard deviation (see Figure 3).

For comparison, Figure 3 also shows the radiance root means square difference (reconstructed-true) obtained with the previous Radiative Transfer Model, σ-SEVIRI. It is seen that $\sigma_{pc}$-SEVIRI has a comparable performance with the previous version. However, $\sigma_{pc}$-SEVIRI is almost 7 times faster than σ-SEVIRI.

### 2.3. Step by Step Description of $\sigma_{pc}$-SEVIRI and Details on the Spectral Data Base to Compute the Monochromatic Predictors, $I_{0}\left(\sigma \right)$, D(σ), and $\tau_{0}\left(\sigma \right)$

**Figure 4:**
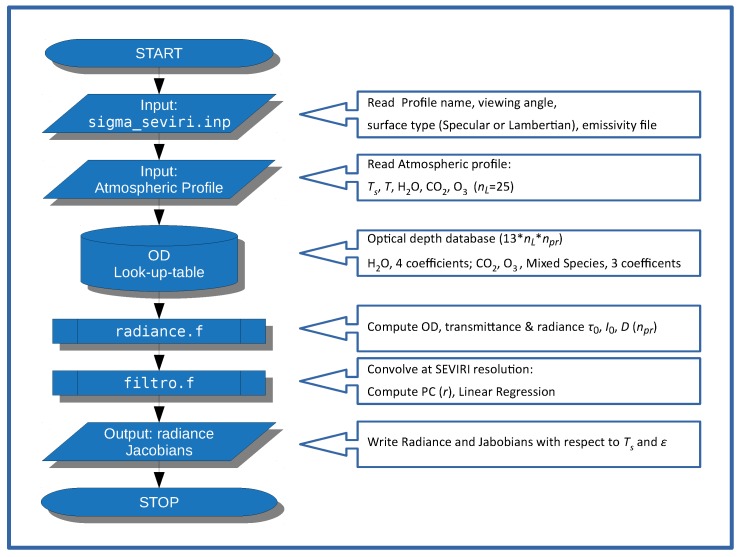
$\sigma_{pc}$-SEVIRI radiative Transfer flow charts.

A detailed step-by-step description of the calculations performed for the $\sigma_{pc}$-SEVIRI forward model is shown in Figure 4. First, the model reads the viewing angle θ, the emissivity file and the type of surface (specular or lambertian). The emissivity file is made up of three values, one for each SEVIRI channel. We stress that the forward module has been optimized to compute SEVIRI radiances for the three atmospheric window channels (e.g., see Table 2).

As a second step, $\sigma_{pc}$-SEVIRI reads surface temperature, $T_{s}$ and atmospheric profiles for T,q,o and CO${}_{2}$. The atmospheric profiles are specified on a pressure grid of $n_{L}=25$ layers (see Table 3). As a default, $T_{s},T,q,o$ are derived from the ECMWF analysis interpolated to the $\sigma_{pc}$-SEVIRI pressure grid and time–space position of the SEVIRI observation. However, $T_{s},T,q,o$ are user-defined parameters, therefore, other choices could be considered.

The next step consists of the calculation of the monochromatic predictors $I_{0}\left(\sigma \right)$, D(σ), and $\tau_{0}\left(\sigma \right)$ for each channel. The spectral database needed for this computations is stored in a monochromatic look-up table of precomputed optical depths. Note that only the spectral information at the predictors wave numbers is stored. As an example, for the channel at 12 μm, the look-up table is organized to perform spectral calculations at 30 specified wave numbers (see e.g., Table 2). The structure and organization of the look-up table is derived from the σ-IASI forward model [23,24,25,26].

For the case at the end of this paper, individual look-tables have been generated for water vapor, carbon dioxide and ozone. The other atmospheric gases form the mixed-gas optical depth and their mixing ratio profile cannot be changed by the user. The reference mixing ratio values for the mixed species are those defined in the AFGL library [31]. The optical depth dependence with respect to the temperature of carbon dioxide, ozone and mixed species are parameterized with second order polynomials. The three polynomial coefficients are stored within the look-up table for each layer and each channel. For water vapour, in order to take into account the self broadening effect [32], the look-up table stores a further coefficient. With this set up, the look-up-table database has the size of 13 (three for CO${}_{2}$, three for O${}_{3}$, three for Mixed Species, four for H${}_{2}$O) times $n_{pr}$ times $n_{L}$ and a storage size of 156 kB.

It should be stressed that the use of a monochromatic look-up table for the calculation of $I_{0}\left(\sigma \right)$, D(σ), and $\tau_{0}\left(\sigma \right)$ simplifies the computation at the various viewing angles. The monochromatic optical depth refers to nadir view. In case of off-nadir view, we have just to scale the computed optical depth to the value of sec(θ) and proceed with the calculation until we have the set of the three predictors, $I_{0}\left(\sigma \right)$, D(σ), and $\tau_{0}\left(\sigma \right)$ at the correct viewing angle.

However, for the computation of radiance and derivative at SEVIRI resolution, we need to use the appropriate angle-dependent couple $\left(U_{\tau },w\right)$, because the final product is non-monochromatic and convolved with the appropriate ISRF. To exemplify the storage needed for the U-basis and w-basis-coefficients, let us assume that we are dealing with the SEVIRI channel at 12 μm. In this case the size of each single U-basis is 30×6, whereas the size of each single *w*-vector is 6. These have to be multiplied by 3 (three parameters, $\langle I_{0}\rangle$, 〈D〉, and $\langle \tau_{0}\rangle$) and by 14 angles. Thus we have a size of 3×14×(30×6+6), which corresponds a storage size of ≈62.5 kB. Considering all channels, we have a final size of ≈192 kB.

Once we have computed the estimates for $\langle I_{0}\rangle$, 〈D〉, and $\langle \tau_{0}\rangle$), the radiance and its derivative are easily computed by using Equations (12)–(14). The executable version of $\sigma_{pc}$-SEVIRI, compiled with the INTEL^®^ compiler *ifort* (v 14.0.1), has a size of 1.5 MB. On a Linux computer with 2.7 GHz CPU it takes 6 ms to compute radiances and derivatives with respect to the surface parameters.

## 3. Data

### 3.1. In Situ Data

**Figure 5:**
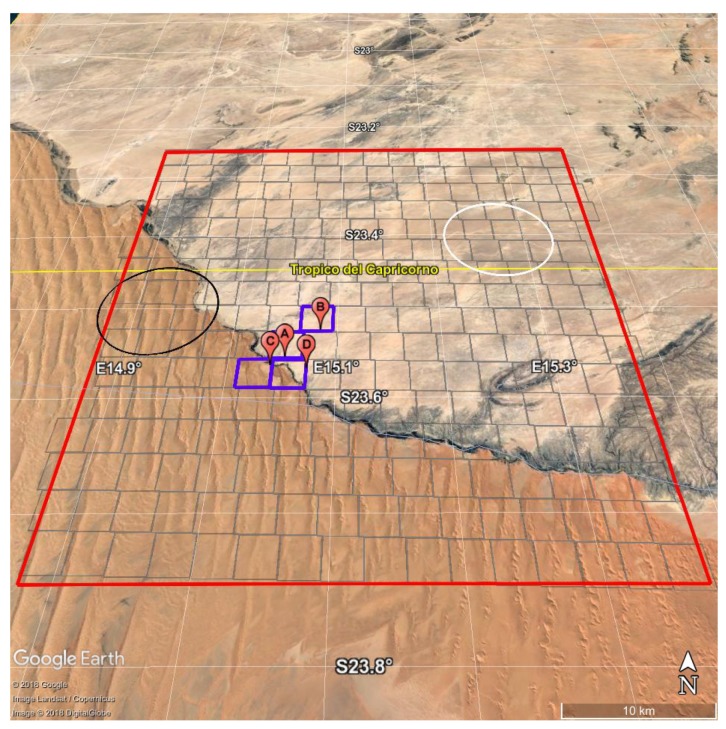
The map shows the target area of this exercise. The red rectangle indicates the SEVIRI target area, whereas the grey line grid-mesh approximates SEVIRI pixels. The red place-marks indicate the position of the ground based measurements and blue rectangles the collocated SEVIRI pixels. White and black circles refers to the IASI footprints used to compare emissivity retrieval.

**Figure 6:**
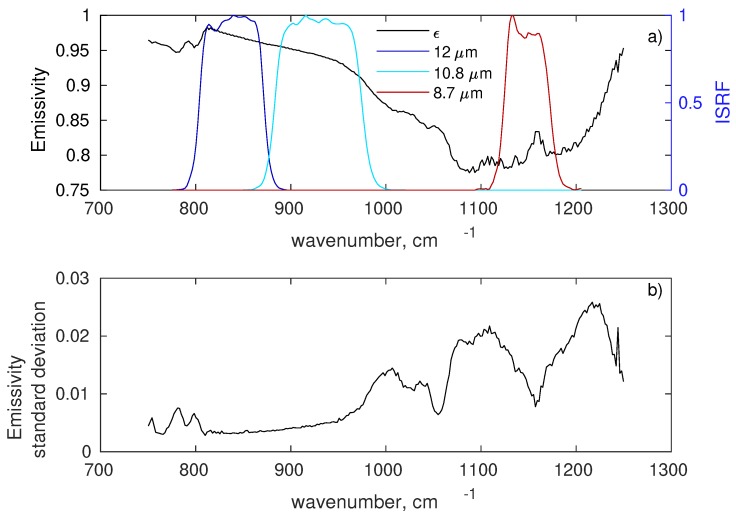
(**a**) Mean in situ emissivity spectrum and the ISRF of the three SEVIRI atmospheric window channels in the Thermal Infrared; (**b**) standard deviation of in situ measurements.

To check the performance of the new $\sigma_{pc}$-SEVIRI, we performed a retrieval exercise over Gobabeb validation station in the Namib desert [22]. SEVIRI observations were acquired, which cover the dates and region of an international LST field inter-comparison experiment. The campaign took place from 17 to 24 June 2017 and the in situ emissivity observations used here for validation purposes were obtained on the Namib gravel plains near Gobabeb LST validation station (Figure 5). Before the experiment, the Namib experienced several years without significant rainfall and the gravel plains were exclusively covered by gravel and sand, i.e., only a minimum amount of dry grass was present. The various measurements performed during the field experiment are described in some detail in [33] and a detailed description of measurement site could be found in [16]. The in-situ emissivity spectra were obtained from measurements with a Fourier Transform Infrared (FTIR) spectrometer by ONERA, the French Aerospace Lab. Each measurement has a footprint with diameter of about 15 cm. The emissivity spectra cover the range from 750 to 1250 cm${}^{-1}$ (8–12 μm) with a spectral resolution of 4 cm${}^{-1}$. This spectral interval exactly matches that of the three SEVIRI channels in the atmospheric window. This is illustrated in Figure 6, which shows the average in situ emissivity spectrum obtained for all available observations. The figure also shows the standard deviation of the measurements in order to assess emissivity variability among the sites shown in Figure 5. The in situ observations are described in Table 4. The absolute uncertainty of each spectral emissivity measurement has been assessed to be about 0.015 in this spectral domain.

### 3.2. IASI Data

For further comparison, we also acquired IASI (Infrared Atmospheric Sounder Interferemeter [34]) observations. In particular, a comparison for two IASI IFOVs was prepared which matches the SEVIRI viewing angles for the area of interest: these two IFOVs are shown in Figure 5.

The region for which SEVIRI emissivity retrievals have been analysed is shown in Figure 5 (between $23.25^{\circ }$ S and $23.75^{\circ }$ S and $14.875^{\circ }$ E and $15.375^{\circ }$ E) and covers an area of almost 50 × 50 km. Inside the region there are 254 SEVIRI pixels (grey mesh in Figure 5), with an approximate view angle (vertical zenith angle) of $34.5^{\circ }$.

Furthermore, IASI data where used to retrieve emissivity as described in [13,16]. The IASI retrievals and the in situ observations have already been analysed and discussed in detail in [16] and a good agreement was found (average spectral difference below 0.025). Here, IASI data are not intended for further comparison with in situ observations, but for a comparison with SEVIRI data. The two IASI observations match the SEVIRI viewing angle and cover two different target areas, one on the gravel plain and the other on the Namib sand dunes.

## 4. Results

### 4.1. SEVIRI v/s In Situ

The in situ emissivity spectra cover the range from 750 to 1250 cm${}^{-1}$ (8–12 μm) with a spectral resolution of 4 cm${}^{-1}$. For the comparison of these spectra with SEVIRI retrieved emissivities, they were convolved with the SEVIRI’s Instrument Spectral Response Function.

**Figure 7:**
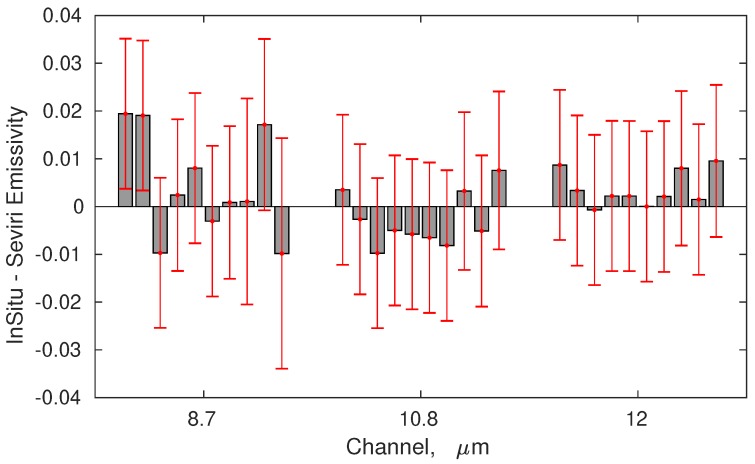
Differences in situ KF retrieval emissivity for the three SEVIRI TIR channels. The comparison includes all in situavailable observations shown in Table 4.

The SEVIRI pixels considered for the comparison are shown in Figure 5. A one by one comparison of the best time–space collocated SEVIRI retrieved emissivity with the ten in situ emissivity spectra described in Table 4 is provided in Figure 7. The figure shows that SEVIRI and in situ emissivities agree within error $\sigma_{\Delta }$ which is computed according to

(24) $$\sigma_{\Delta }={\left(\sigma_{r}^{2}+\sigma_{s}^{2}\right)}^{\frac{1}{2}}$$

where $\sigma_{r}^{2}$ is the variance of the retrieved SEVIRI emissivities and $\sigma_{s}$ is the the in situ error propagated to SEVIRI’s spectral resolution.

**Figure 8:**
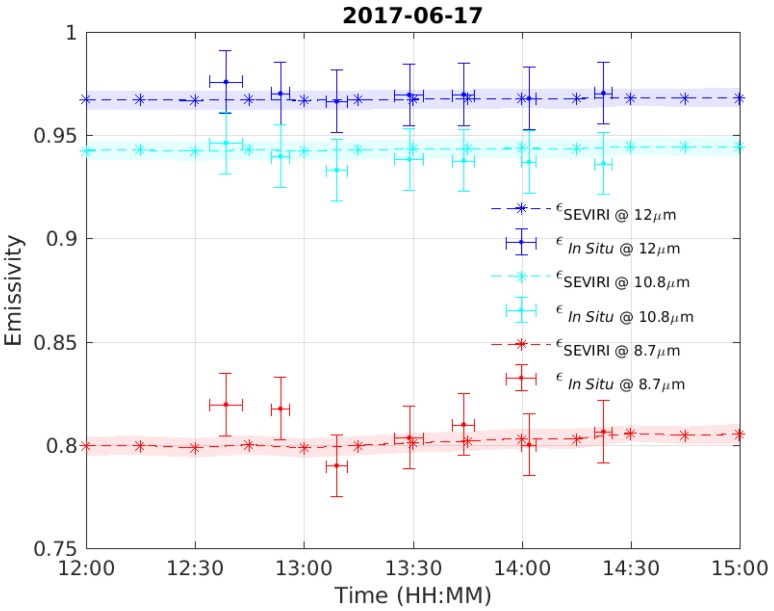
Comparison between SEVIRI and in situ emissivity for 17 June 2017. The shaded area gives the ±1σ confidence interval of the retrieval. The horizontal bar for the in situ observations correspond to the time taken to acquire a single measurement.

For the 17 June 2017, we have a set of 7 consecutive in situ observations can be compared with the SEVIRI retrieved time sequence. The comparison is provided in Figure 8. To better understand and interpret the comparison, it has to be stressed that the SEVIRI observations refer to pixel A in Figure 5 and correspond to different acquisition times. In situ observations not only correspond to different times, but also to various locations, although all locations are within the same SEVIRI pixel A (see Figure 8). Therefore, Figure 8 also provides some information on the sub-pixel variability of emissivity: as expected, variability is more marked at 8.7 μm because of its dependence of quartz soil content. However, overall, there is high consistency between in situobservations and SEVIRI retrieved emissivities.

**Figure 9:**
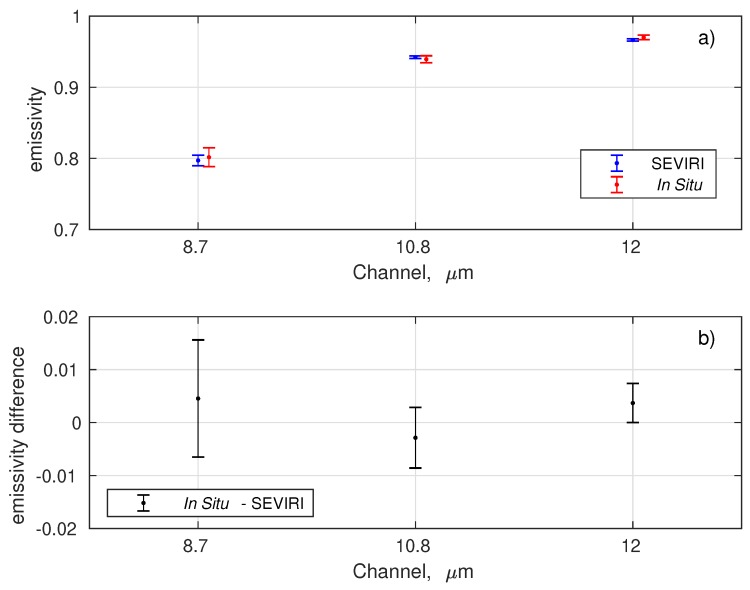
(**a**) Comparison between SEVIRI emissivity and in situ measurements. Error bars represent the standard deviation (variability) of retrievals and in situ measurements. (**b**) Emissivity difference (in-situ-retrieved).

To reduce random variability, Figure 9 shows the comparison between SEVIRI retrieved emissivity and in situ emissivity, but averaged over time and space. The error bar in Figure 9 is the standard deviation of these observations. For the in situ observations, the emissivity in Figure 9 has been averaged over all available measurements and the error bar is the standard deviation of these measurements. From Figure 9, we see that there is a very high consistency between SEVIRI and in situ emissivities. In fact, the mean difference is below 0.01.

### 4.2. SEVIRI v/s IASI

During the night of 22 July 2017 at 21:00:03 IASI observations overlapped the target area in Figure 5. In particular, one observation with IFOV angle of $25.44^{\circ }$ covered an area over desert dunes (black circle in Figure 5); a second IASI observation with IFOV angle of $27.86^{\circ }$ was located over the gravel plain (white circle in Figure 5).

In the following comparison, SEVIRI emissivity retrievals at 21:00:00 GMT have been considered: for the sand dunes nine SEVIRI pixels inside the IASI IFOV were averaged while for the gravel plain 10 SEVIRI pixels overlapped with the IASI IFOV.

IASI spectral emissivity has been retrieved and reduced to SEVIRI spectral resolution by convolving it with the SEVIRI ISRF. In this way, we computed SEVIRI-like emissivity for IASI.

**Figure 10:**
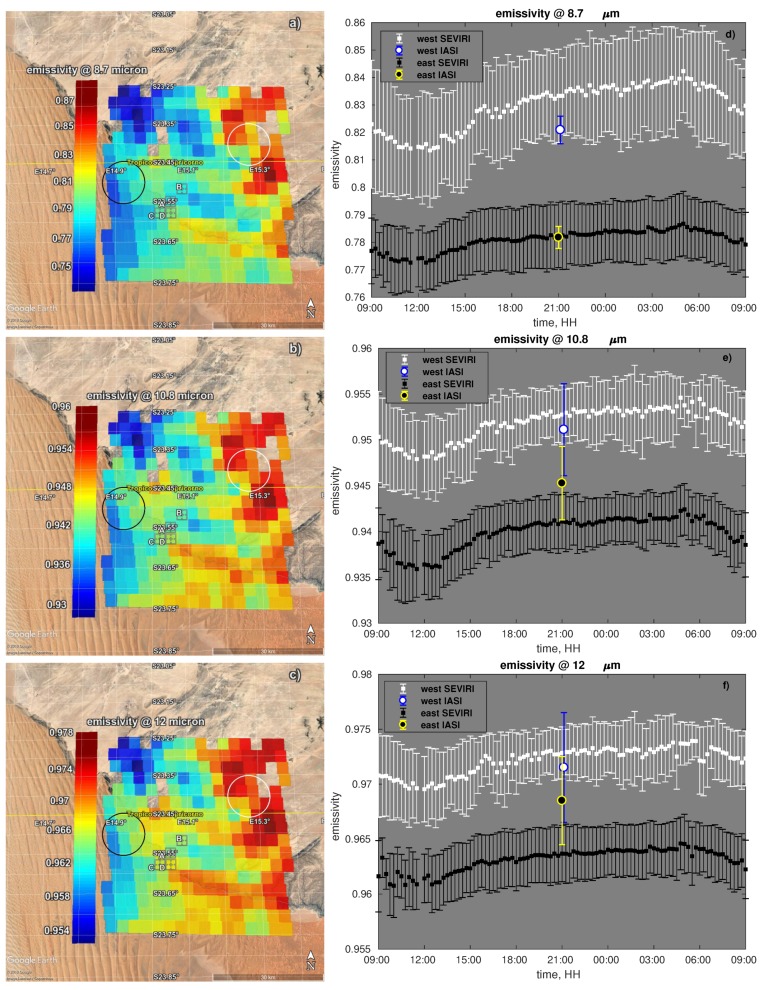
SEVIRI vs IASI emissivity. Panels on the left show the map of SEVIRI retrieved emissivity at 21:00:00 of 22 June 2017. Panels on the right show IASI retrieved emissivity and a time series of co-located SEVIRI retrieved emissivity. Panels (**a**,**d**) refer to the channel at 8.7 μm; panels (**b**,**e**) to the channel at 10.8 μm; panels (**c**,**f**) to the channel at 12 μm. In panels (**d**–**f**) SEVIRI dots and related error bars represent respectively the mean and the standard deviation of the retrieved emissivity for the SEVIRI pixels inside IASI footprint respectively. The IASI errorbar is the root mean square error of the retrieval computed based on the corresponding emissivity error covariance matrix.

The comparison is illustrated in Figure 10 where the left panels show SEVIRI retrieved emissivity at 21:00:00 at 8.7 μm (panel a), at 10.8 μm (panel b) and at 12 μm (panel c). The right panels show the IASI-retrieved emissivities superimposed on the time series of co-located SEVIRI retrievals.

We stress that IASI and SEVIRI retrieved emissivity both show lower values for the western pixels than the eastern ones. This behaviour is in agreement with the surface characteristics, i.e., sand v/s gravel.

SEVIRI time series for both footprints show a minimum around noon and a maximum during night. These day-night variations are consistent with the findings of other authors (e.g., [7]) and follow the daily cycle of soil moisture.

IASI and SEVIRI emissivities show good agreement that is better over the gravel plain than the sand dunes. This can be understood when considering that the gravel plain is a flat area, whereas the sand dunes cover an area with varying slope, which can complicate the observing geometry.

## 5. Conclusions

A new radiative transfer model specifically developed for the retrieval of surface emissivity and temperature from SEVIRI infrared channels at 8.7, 10.8, 12 μm has been developed. The forward model has been specifically optimized to provide radiances at the three channels with improved computational speed. In effect, the new model is about seven times faster than the previous version.

The forward model has been embedded in a Kalman Filter algorithm for the time-continuous retrieval of surface temperature and emissivity. A retrieval exercise has been set up and performed for a region located close to Gobabeb validation station in the Namib desert. Emissivity retrievals have been compared with in situ measurements as well as with satellite observations obtained from IASI.

We have shown that the retrieved emissivities compare well with in situ observations. Furthermore, in situ observations show a sub-pixel variability, which highlights the intrinsic difficulty of validation of satellite-based emissivity products. As expected, the variability is larger at 8.7 μm because of the sensitivity of this channel to the soil’s content of quartz. However, spatial averaging improves the comparison, and differences are reduced to ±0.01. A consistent behaviour is also seen between e IASI and SEVIRI emissivities.

Our forward modelling approach preserves many advantages related to mono-chromatic radiance calculations, such as applicability to other instruments by simply changing the instrument spectral response function, independence of viewing geometry. If the ISRF is changed, only the regression coefficients need to be updated, but the monochromatic look-up table remains unchanged.

In summary, our approach combines the accuracy of monochromatic radiative transfer with the speed of polychromatic forward modelling, which opens the way to accurate real time physically based retrieval of surface emissivity and temperature from SEVIRI.

## Figures and Tables

**Table 1:** SEVIRI infrared channels and radiometric noise performance in NEDT (noise Equivalent Difference Temperature).

Channel #	Wave Length Center (μm)	Wave Number Center (cm${}^{-1}$)	Radiometric Noise (NEDT, K)
4	3.9	2564	0.35 at 300 K
5	6.2	1613	0.75 at 250 K
6	7.3	1370	0.75 at 250 K
7	8.7	1148	0.28 at 300 K
8	9.7	1035	1.5 at 255 K
9	10.8	929	0.25 at 300 K
10	12.0	838	0.37 at 300 K
11	13.4	746	1.80 at 270 K

**Table 2:** The table summarizes the number of predictors in radiance space ($n_{pr}$), principal components space (*r*) and the variance explained in percentage units ($\eta^{2}$). The coefficients $b_{0},b_{1}$ are also shown.

Channel (μm)	$n_{\mathrm{pr}}$	*r*	$\eta^{2}$ %	$\sigma_{0}$ (cm${}^{-1}$)	$b_{1}$	$b_{0}$ (W/m${}^{2}$ (cm${}^{-1}$)${}^{-1}$ sr${}^{-1}$ K${}^{-1}$)
12	30	6	99.95	838	0.99799	1.03182 × 10${}^{-6}$
10.8	20	5	99.94	929	0.99376	6.56584 × 10${}^{-6}$
8.7	30	9	99.95	1148	1.00304	−2.55525 × 10${}^{-6}$
Total	80	20				

**Table 3:** Pressure layering of the atmosphere used in σ-SEVIRI.

Layer	Pressure (hPa)	Layer	Pressure (hPa)	Layer	Pressure (hPa)	Layer	Pressure (hPa)
1	1050.0–975.0	8	650.0–550.0	15	125.0–85.0	22	6.0–4.0
2	975.0–937.5	9	550.0–450.0	16	85.0–60.0	23	4.0–2.5
3	937.5–912.5	10	450.0–350.0	17	60.0–40.0	24	2.5–1.5
4	912.5–875.0	11	350.0–275.0	18	40.0–25.0	25	1.5–0.5
5	875.0–825.0	12	275.0–225.0	19	25.0–15.0		
6	825.0–750.0	13	225.0–175.0	20	15.0–8.5		
7	750.0–650.0	14	175.0–125.0	21	8.5–6.0		

**Table 4:** Description of the ground based emissivity measurements.

Date	Letter	Area	Number of Aggregated Measurements	Comments
17 June 2017	A	Mast	7	seven spots around the fence, shared with Themacs
				two measurements per spot
22 June 2017	B	Mast 2	1	15 measurements along a 20-m line, four on disturbed soil
				measurements (the gravel is covered by sand/dust)
23 June 2017	C	GRTC	1	16 measurements, three sets of samples; 30 m between sets,
				each set covering a 5-m line
24 June 2017	D	Road	1	ten measurements along a 30-m line at the starting
				point of the road experiment

## Abbreviations

The following abbreviations are used in this manuscript:

AVHRR	Advanced Very High Resolution Radiometer
ECMWF	European Centre for Medium Range Weather Forecasts
EUMETSAT	European Centre for the Exploitation of Meteorological Satellites
ESA	European Space Agency
IASI	Infrared Atmospheric Sounder Interferometer
LSA	Land Surface Analysis
MIUR	Italian Ministry of Education, University and Research
NPL	National Physical Laboratory
PCA	Principal Component Analysis
SAF	Satellite Applictaion Facility
